# An Exploratory Study of Spectroscopic Glutamatergic Correlates of Cortical Excitability in Depressed Adolescents

**DOI:** 10.3389/fncir.2016.00098

**Published:** 2016-11-29

**Authors:** Charles P. Lewis, John D. Port, Mark A. Frye, Jennifer L. Vande Voort, Stephanie H. Ameis, Mustafa M. Husain, Zafiris J. Daskalakis, Paul E. Croarkin

**Affiliations:** ^1^Mayo Clinic Depression Center, Department of Psychiatry and Psychology, Mayo ClinicRochester, MN, USA; ^2^Department of Radiology, Mayo ClinicRochester, MN, USA; ^3^Faculty of Medicine, Department of Psychiatry, University of TorontoToronto, ON, Canada; ^4^Temerty Centre for Therapeutic Brain Intervention, Campbell Family Mental Health Research Institute, Centre for Addiction and Mental Health, University of TorontoToronto, ON, Canada; ^5^The Margaret and Wallace McCain Centre for Child, Youth and Family Mental Health, Campbell Family Mental Health Research Institute, The Centre for Addiction and Mental Health, University of TorontoToronto, ON, Canada; ^6^Department of Psychiatry, University of Texas Southwestern Medical CenterDallas, TX, USA; ^7^Department of Neurology and Neurotherapeutics, University of Texas Southwestern Medical CenterDallas, TX, USA; ^8^Department of Psychiatry and Behavioral Sciences, Duke University School of MedicineDurham, NC, USA

**Keywords:** transcranial magnetic stimulation, proton magnetic resonance spectroscopy, depression, child and adolescent, cortical excitability, glutamate

## Abstract

**Introduction**: Transcranial magnetic stimulation (TMS) research has suggested dysfunction in cortical glutamatergic systems in adolescent depression, while proton magnetic resonance spectroscopy (^1^H-MRS) studies have demonstrated deficits in concentrations of glutamatergic metabolites in depressed individuals in several cortical regions, including the anterior cingulate cortex (ACC). However, few studies have combined TMS and MRS methods to examine relationships between glutamatergic neurochemistry and excitatory and inhibitory neural functions, and none have utilized TMS-MRS methodology in clinical populations or in youth. This exploratory study aimed to examine relationships between TMS measures of cortical excitability and inhibition and concentrations of glutamatergic metabolites as measured by ^1^H-MRS in depressed adolescents.

**Methods**: Twenty-four adolescents (aged 11–18 years) with depressive symptoms underwent TMS testing, which included measures of the resting motor threshold (RMT), cortical silent period (CSP), short-interval intracortical inhibition (SICI), and intracortical facilitation (ICF). Fourteen participants from the same sample also completed ^1^H-MRS in a 3 T MRI scanner after TMS testing. Glutamate + glutamine (Glx) concentrations were measured in medial ACC and left primary motor cortex voxels with a TE-optimized PRESS sequence. Metabolite concentrations were corrected for cerebrospinal fluid (CSF) after tissue segmentation. Pearson product-moment and Spearman rank-order correlations were calculated to assess relationships between TMS measures and [Glx].

**Results**: In the left primary motor cortex voxel, [Glx] had a significant positive correlation with the RMT. In the medial ACC voxel, [Glx] had significant positive correlations with ICF at the 10-ms and 20-ms interstimulus intervals (ISIs).

**Conclusion**: These preliminary data implicate glutamate in cortical excitatory processes measured by TMS. Limitations included small sample size, lack of healthy control comparators, possible age- and sex-related effects, and observational nature of the study. Further research aimed at examining the relationship between glutamatergic metabolite concentrations measured through MRS and the excitatory and inhibitory physiology measured through TMS is warranted. Combined TMS-MRS methods show promise for future investigations of the pathophysiology of depression in adults as well as in children and adolescents.

## Introduction

Depression is common in youth, affecting 5.7% of US adolescents aged 12–17 (Pratt and Brody, 2014). However, the neurobiological mechanisms of depression in this age group remain inadequately understood and may differ from those in adults, as numerous systems with putative roles in the pathophysiology of depression undergo significant developmental changes (Zalsman et al., 2006). Several converging lines of neurophysiological and neurochemical evidence point to glutamate and γ-aminobutyric acid (GABA), respectively the mammalian brain’s predominant excitatory and inhibitory neurotransmitters, as central to the etiology of depression (Krystal et al., 2002; Sanacora and Saricicek, 2007; Levinson et al., 2010; Croarkin et al., 2011). Transcranial magnetic stimulation (TMS) involves the induction of electric current in the cerebral cortex by a focused magnetic field, which permits noninvasive, *in vivo* investigation of excitatory and inhibitory neural circuits mediated by glutamate and GABA. Studies utilizing pharmacologic agents with known effects at specific neurotransmitter receptors have helped to elucidate the mechanisms of various single- and paired-pulse TMS paradigms (Ziemann et al., 1996a, 2015; Siebner et al., 1998; Werhahn et al., 1999). These include measures of cortical excitability, such as the resting motor threshold (RMT) and intracortical facilitation (ICF), as well as indices of cortical inhibitory processes, such as the cortical silent period (CSP), short-interval intracortical inhibition (SICI), and long-interval intracortical inhibition (LICI; Hanajima and Ugawa, 2008; Sandbrink, 2008; Wolters et al., 2008; Ziemann et al., 2015). TMS has been utilized to investigate disturbances in excitatory and inhibitory systems in a range of psychopathology in adults, including depression (Steele et al., 2000; Bajbouj et al., 2006; Lefaucheur et al., 2008; Levinson et al., 2010). A meta-analysis of adult studies found reductions in CSP duration and reduced SICI in adults with major depressive disorder (MDD) compared to healthy controls, although no consistent differences in RMT or ICF (Radhu et al., 2013). Few studies have examined TMS measures of cortical inhibition and facilitation in children and adolescents with depression. However, our group previously found increased ICF, but no differences in inhibitory measures, in children and adolescents with MDD compared to healthy controls (Croarkin et al., 2013). In a *post hoc* analysis of the same sample, measures of depression severity showed significant correlations with CSP duration as well as ICF (Lewis et al., 2016). These preliminary findings suggest that children and adolescents with depression may have distinct excitatory and inhibitory cortical physiology compared to depressed adults.

**Figure 1:**
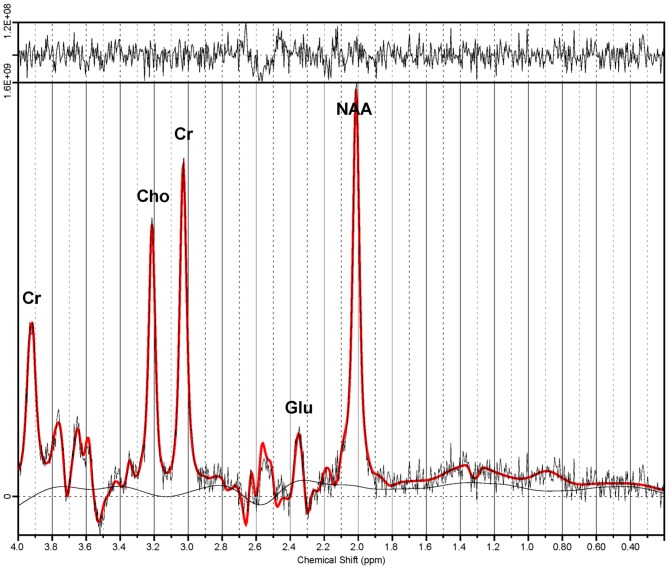
**Proton magnetic resonance spectrum from the anterior cingulate cortex (ACC).** Spectroscopic data were acquired via a TE-optimized PRESS sequence (TE 80 ms) at 3 T. Quantitative analysis was performed by LCModel (Provencher, 1993). Note the dominant glutamate (Glu) peak at 2.34 ppm. Also shown are signal peaks of choline (Cho), creatine (Cr) and *n*-acetylaspartate (NAA).

Proton magnetic resonance spectroscopy (^1^H-MRS) is a magnetic resonance imaging (MRI) modality that allows quantification of various compounds in live tissue, including brain (Figure 1). Glutamate (Glu) and its related metabolite glutamine (Gln) have overlapping peaks in the ^1^H-MR spectrum. Consequently, the sum of their MR signals (Glx), which also incorporates minor signal contributions from GABA and glutathione, is often reported (Maddock and Buonocore, 2012). Multiple prior studies examined ^1^H-MRS measures of excitatory and inhibitory neurochemistry in adults with mood disorders (Yildiz-Yesiloglu and Ankerst, 2006; Yüksel and Öngür, 2010). In adult studies, patients with MDD had reductions in glutamatergic metabolites (Glx, Glu, or both) compared to healthy controls in the anterior cingulate cortex (ACC; Auer et al., 2000; Pfleiderer et al., 2003; Hasler et al., 2007; Horn et al., 2010; Merkl et al., 2011; Portella et al., 2011), other prefrontal cortical regions (Michael et al., 2003a; Hasler et al., 2007) and anterior temporal structures (Michael et al., 2003b; Block et al., 2009). Conversely, in one study glutamate levels were elevated in the occipital cortex of depressed adults (Sanacora et al., 2004). Additionally, cortical decrements in ^1^H-MRS-measured glutamatergic metabolites have been shown to normalize with remission (Hasler et al., 2005; Taylor et al., 2009) and with response to treatment (Michael et al., 2003a,b; Pfleiderer et al., 2003). Several ^1^H-MRS studies have investigated glutamatergic abnormalities in depressed, medication-naïve children and adolescents (Kondo et al., 2011), finding decreases in concentrations of Glx (Mirza et al., 2004; Rosenberg et al., 2004) and Glu (Rosenberg et al., 2005) in the ACC, but no decreases glutamatergic metabolites in the occipital cortex (Mirza et al., 2004; Rosenberg et al., 2005), when compared to healthy control youth. In summary, ^1^H-MRS studies to date have yielded substantial evidence of glutamatergic disturbances in adults with unipolar depression, particularly in the ACC, while several adolescent studies have indicated a similar pattern of altered glutamatergic neurochemistry in depression.

The use of neurophysiologic TMS paradigms in conjunction with MRS permits the assessment of relationships between cortical circuitry functioning and brain neurochemistry (Bestmann and Feredoes, 2013). However, to date this promising approach has seldom been utilized. Two previous studies have examined relationships between TMS measures of cortical excitability and inhibition and ^1^H-MRS-measured glutamate in healthy adults. Stagg et al. (2011) found that the slope of the motor evoked potential (MEP) input-output (I-O) curve, a TMS measure of excitability, correlated strongly with ^1^H-MRS-measured [Glu] and [GABA] in the corresponding primary motor cortex. By contrast, Tremblay et al. (2013) found a positive correlation between motor cortex [Glx] and CSP duration (which persisted when controlling for GABA concentration), but the authors found no correlation between ^1^H-MRS-measured [GABA] and CSP, SICI, or LICI. Few studies have utilized combined TMS-MRS methods in psychiatric populations, and those that have done so typically examined treatment-induced changes in excitatory and inhibitory neurochemistry following repetitive transcranial magnetic stimulation (rTMS) for depression (Croarkin et al., 2016; Dubin et al., 2016). To our knowledge, no prior studies, either adult or pediatric, have examined the associations between TMS measures of excitatory and inhibitory neurophysiology and MRS-measured glutamatergic neurochemistry in depressed individuals. The current exploratory study aimed to investigate relationships between concentrations of ^1^H-MRS-measured glutamatergic metabolites (Glx) and TMS measures of cortical excitability (RMT, ICF) and inhibition (CSP, SICI) in a sample of depressed adolescents. For ^1^H-MRS, a left primary motor cortical voxel directly under the location of the TMS coil was selected to examine glutamatergic metabolites in the stimulated region of cortex. Another voxel was selected in the ACC, a structure that has complex roles in the pathophysiology of mood disorders, is often reliably studied with ^1^H-MRS, and has numerous motor projections (Devinsky et al., 1995; Paus, 2001). We anticipated that TMS measures of glutamate-mediated excitability (particularly ICF) would correlate directly with ^1^H-MRS-measured [Glx] in both the ACC and motor cortex.

## Materials and Methods

### Study Design and Overview

This was a cross-sectional study of depressed, treatment-seeking adolescents. All participants underwent diagnostic and clinical assessments and TMS neurophysiology measures. A subset of eligible participants underwent MRI/MRS. The TMS measures were obtained during a single session, and MRI/MRS was subsequently performed on the same day. No treatment was provided as part of the study protocol, but following completion, participants were referred for additional care if clinically appropriate. Prior to enrollment, the study protocol and all procedures were approved by the Mayo Clinic institutional review board. Participants under the age of 18 years granted written assent, and parents or legal guardians gave written informed consent. Participants who were 18 years of age provided written consent.

### Participants

The sample was comprised of 24 participants, aged 11–18 years, with clinically significant depressive symptoms. All participants were right-handed as determined by the Edinburgh Inventory (Oldfield, 1971). Additionally, all participants were fluent in the English language, and at least one parent or guardian also was fluent in English. Exclusion criteria included a primary psychiatric diagnosis other than a depressive disorder; history of unprovoked seizures, febrile seizures, seizure disorder or family history of epilepsy; significant positives on the TMS Adult Safety Screen (Keel et al., 2001); implanted metal in the head or other contraindications to MRI/MRS (of note, adolescents were eligible for participation in the study if they had metallic orthodontic hardware, although they were excluded from the MRI/MRS portion of the protocol); prior brain surgery or risk for increased intracranial pressure; pregnancy or suspected pregnancy; or other unstable medical conditions. Additionally, participants at risk of imminent self-harm or suicide as determined by a study psychiatrist (CPL, JLVV, PEC) were not eligible for participation.

### Depression Severity Measures

Participants’ depressive symptoms were assessed with two instruments. The Children’s Depression Rating Scale—Revised (CDRS-R; Poznanski et al., 1984) is a 17-item, clinician-rated scale that incorporates both parent- and adolescent-reported data into composite scores for specific depressive symptoms. The CDRS-R clinician total score is reported. The Quick Inventory of Depressive Symptomatology—Adolescent (17 Item)—Self-Report (QIDS-A_17_-SR) is a self-report version of the clinician-rated QIDS-A_17_-C (Bernstein et al., 2010), a 17-item assessment of depressive symptoms. The QIDS- A_17_-SR total score is reported.

### TMS Procedures and Measures

All participants underwent TMS to assess cortical inhibition and excitability. Single- and paired-pulse stimulation paradigms were applied to the left primary motor cortex, and measures were obtained via surface electromyography (EMG) of the contralateral (right) abductor pollicis brevis (APB) muscle during TMS stimulation. Participants and research personnel wore earplugs throughout the procedure.

**Figure 2:**
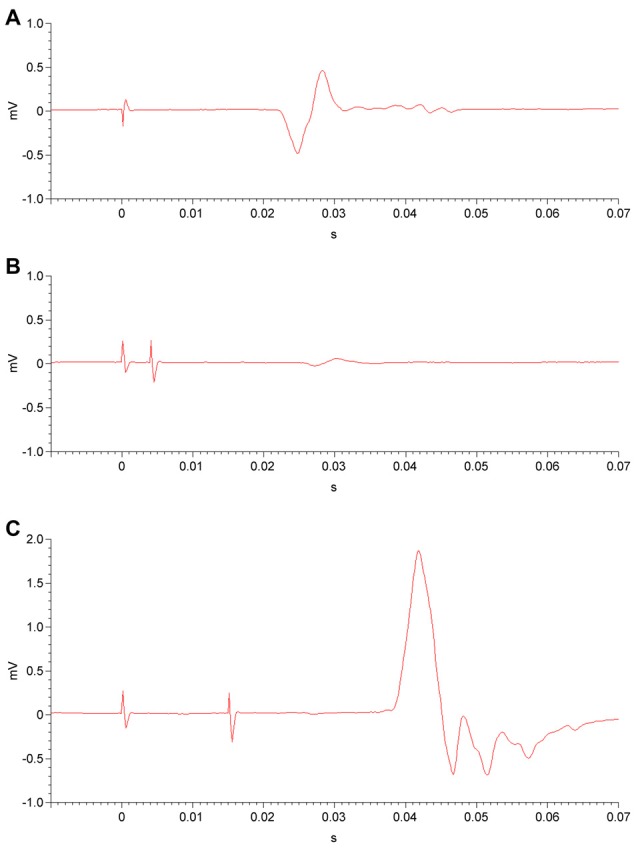
**Electromyography (EMG) of unconditioned, inhibited and facilitated motor evoked potentials (MEPs). (A)** Following a single transcranial magnetic stimulation (TMS) pulse (at time = 0) to the primary motor cortex at an intensity above the resting motor threshold (RMT), an MEP is detected by EMG in the corresponding muscle. **(B)** In the short-interval intracortical inhibition (SICI) paradigm, a subthreshold conditioning stimulus (at time = 0) is administered to the primary motor cortex. After a 2-ms or 4-ms interstimulus interval (4-ms shown), a second, suprathreshold test stimulus is delivered. The resulting MEP is diminished in amplitude, i.e., inhibited. **(C)** In the intracortical facilitation (ICF) paradigm, a subthreshold conditioning stimulus (at time = 0) and a subsequent suprathreshold test stimulus are administered to the primary motor cortex, separated by a 10-ms, 15-ms, or 20-ms interstimulus interval (15-ms shown). This results in an MEP that is facilitated, or increased in amplitude.

Two Magstim 200 stimulators with a BiStim module (Magstim Co. Ltd., Whitland, Wales, UK) and a figure-of-eight electromagnetic coil (70 mm diameter for each coil loop) were used. The coil was positioned tangentially on the surface of the scalp, superficial to the left primary motor cortex. In order to locate the optimal coil position, the position was adjusted in 1-cm increments while stimulating with single magnetic pulses and observing for the maximal contraction of the contralateral APB while the muscle was at rest. After determining the optimal position of the coil for APB stimulation, pulse intensity was increased gradually until the MEP on EMG reached at least 50 microvolts in 5 of 10 trials (Figure 2A); the intensity at which this occurred was defined as the RMT (Rossini et al., 2015). After establishing the RMT, other single- and paired-pulse measures were acquired.

Paired-pulse measures involved the application of two paired magnetic pulses in rapid succession to the left primary motor cortex, again while the APB was at rest. For both SICI and ICF paradigms, a subthreshold conditioning stimulus (80% of RMT) was followed by a suprathreshold test stimulus (calibrated to result in an MEP with a peak-to-peak amplitude of 1 mV on EMG). The interstimulus intervals (ISIs) between conditioning and test stimuli were 2 ms and 4 ms for the SICI paradigm (Figure 2B) and were 10 ms, 15 ms, and 20 ms for the ICF paradigm (Figure 2C). For both SICI and ICF, the amplitude of the conditioned MEP occurring after the test stimulus is expressed as a ratio to the mean unconditioned MEP amplitude. Paired-pulse measures were obtained in a randomized and counterbalanced fashion, with a total of twelve trials at each ISI. MEP amplitude ratios were averaged for each ISI.

**Figure 3:**
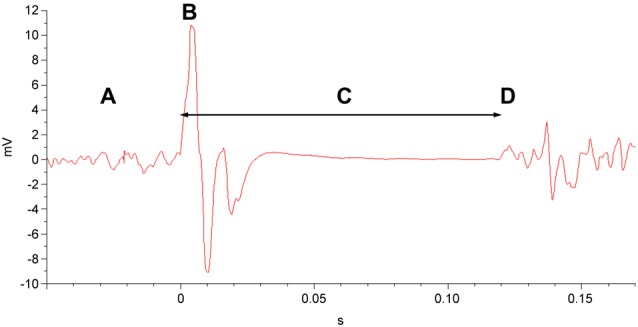
**EMG of the cortical silent period (CSP).** In a muscle exhibiting tonic motor activity **(A)**, a single TMS pulse at 140% of the RMT **(B)** is delivered to the corresponding area of the primary motor cortex. This is followed by the CSP **(C)**, a quiescent interval that ends with resumption of motor activity **(D)**.

To measure the CSP duration, participants maintained tonic, voluntary contraction of the right APB (at 20% of maximum contraction strength, measured by hand-held dynamometer), during which single magnetic pulses at 140% of the RMT were applied to the left primary motor cortex. The CSP was recorded via EMG of the right APB following each pulse (Figure 3). CSP duration was averaged over the 10 trials performed.

At the conclusion of the TMS session, the position of the center of the TMS coil was marked with a vitamin E capsule, affixed to the scalp with adhesive, as a point of reference for the primary motor cortex during the ^1^H-MRS scan.

#### TMS Measures of Cortical Inhibition

Two TMS measures that index GABAergic cortical inhibition were obtained. SICI is induced by paired TMS pulses: an initial, subthreshold conditioning stimulus followed 1–5 ms later by a suprathreshold test stimulus, with a resultant MEP that is diminished in amplitude compared to an unconditioned MEP (Kujirai et al., 1993; Ziemann et al., 1996b; Fisher et al., 2002; Ilić et al., 2002). Prior research has implicated intracortical GABAergic inhibitory circuits in the origins of SICI (Di Lazzaro et al., 1998; Hanajima et al., 1998, 2003). Previous TMS-pharmacologic studies have indicated that SICI results from GABA_A_ receptor-mediated neurotransmission in the motor cortex (Di Lazzaro et al., 2000, 2005; Ilić et al., 2002; Ziemann, 2004; Hanajima and Ugawa, 2008; Ziemann et al., 2015), specifically those GABA_A_ receptors containing α2 or α3 subunits (Di Lazzaro et al., 2007).

The CSP is an interval following a single suprathreshold TMS pulse during which the EMG is quiescent, indicating an interruption in voluntary motor activity (Day et al., 1989a,b; Cantello et al., 1992). The CSP can persist as long as 300 ms, and while spinal inhibitory neurons are thought to contribute to the first 50–75 ms, the latter portion of the CSP is posited to be produced by inhibition of motor cortical origin (Day et al., 1989b; Fuhr et al., 1991; Wolters et al., 2008; Ziemann et al., 2015). Studies involving pharmacologic agents with known effects on GABA receptors have implicated activation of both GABA_A_ and GABA_B_ receptors in the production of the CSP (Werhahn et al., 1999; Ziemann et al., 2015).

#### TMS Measures of Cortical Excitability

By convention, the motor threshold is defined as the lowest magnetic stimulus intensity at which an MEP is reliably produced on EMG in the target muscle (Rossini et al., 2015). The motor threshold is considered to represent excitability of cortico-cortical neurons and their synapses onto corticospinal neurons (Ziemann et al., 2015). The RMT is believed to index glutamatergic synaptic activity more specifically than the active motor threshold (AMT), which may depend to a greater extent on corticospinal and spinal axonal excitability (Paulus et al., 2008). Di Lazzaro et al. (2003) demonstrated that the *N*-methyl-D-aspartate (NMDA) glutamatergic antagonist ketamine causes dose-dependent reductions in the RMT, possibly through enhancement of non-NMDA, α-amino-3-hydroxy-5-methyl-4-isoxazolepropionic acid (AMPA) receptor-mediated fast glutamatergic neurotransmission (Paulus et al., 2008; Ziemann et al., 2015). However, other NMDA antagonists such as memantine (Schwenkreis et al., 1999) and dextromethorphan (Ziemann et al., 1998) have not demonstrated effects on the RMT. The receptor-nonspecific antiglutamatergic medication riluzole has been shown to increase RMT over time (Schwenkreis et al., 2000). As such, RMT is thought to depend largely on a combination of both NDMA receptor and AMPA receptor-mediated neurotransmission.

Like SICI, ICF involves pairing a subthreshold conditioning stimulus with a suprathreshold test stimulus, albeit with a slightly longer ISI of 7–20 ms. However, pairing TMS pulses in this longer ISI range leads to MEPs that are increased in amplitude (i.e., facilitated) compared to unconditioned MEPs (Kujirai et al., 1993; Ziemann et al., 1996b, 2015; Di Lazzaro et al., 2006; Hanajima and Ugawa, 2008). It has been proposed that ICF originates in the motor cortex as opposed to spinal neurons, and it is hypothesized to be caused by a population of cortical interneurons distinct from those responsible for SICI (Ziemann et al., 1996b; Hanajima et al., 1998), although its mechanisms may be more complex than those of SICI (Di Lazzaro et al., 2006). TMS-pharmacological research has shown the ICF effect to vary with agents that act at GABA_A_ receptors (Ziemann et al., 1996a) as well as with medications acting on NMDA receptors (Liepert et al., 1997; Ziemann et al., 1998; Schwenkreis et al., 1999) and by other glutamatergic mechanisms (Schwenkreis et al., 2000). Although ICF is a complex phenomenon in which several neurotransmitters are involved, it has been proposed as an index of cortical excitability mediated predominantly by glutamatergic neurotransmission (Hanajima and Ugawa, 2008; Ziemann et al., 2015).

### MRS Procedures and Measures

#### MRI and ^1^H-MRS Acquisition

Participants underwent structural MRI and TE-optimized proton MRS using a method similar to that published previously (Croarkin et al., 2016). Briefly, scans were obtained using a General Electric 3 T Discovery MRI scanner (GE Medical Systems, Inc., Waukesha, WI, USA) equipped with an 8-channel head coil. Volumetric data for the cerebrospinal fluid (CSF) correction were acquired using a FAST 3D SPGR sequence (sagittal acquisition, TR = 7.4 ms, TE = 3.0 ms, flip angle = 8°, voxel 1.02 mm^3^ × 1.02 mm^3^ × 1.2 mm^3^). Structural MRI scans were reviewed by a neuroradiologist (JDP) for incidental findings.

**Figure 4:**
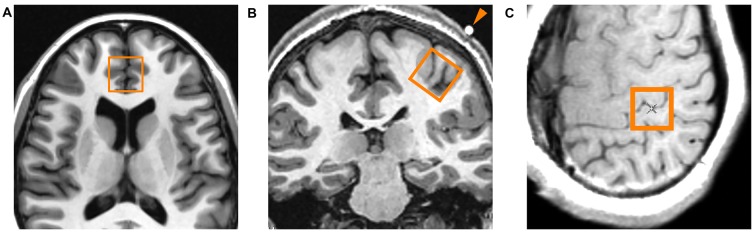
**Location of proton magnetic resonance spectroscopy (^1^H-MRS) voxels. (A)** Axial localizer slice indicating the location of the midline anterior cingulate voxel. This voxel encompasses the pregenual ACC of both cerebral hemispheres. **(B)** Coronal localizer slice indicating the location of the left primary motor cortex voxel. The arrowhead indicates the vitamin E capsule which was placed directly over the abductor pollicis brevis (APB) point as determined by TMS. **(C)** Axial oblique localizer slice showing another view of the left primary motor cortex voxel. The center of the voxel is located immediately beneath the vitamin E capsule, encompassing the hand knob area of the primary motor cortex.

A systematic approach to voxel positioning was followed for all participants (Figure 4). During the TMS procedure, a vitamin E capsule was affixed to the scalp overlying the left APB point. For the left primary motor cortex voxel, a set of axial oblique localizer slices were placed parallel to the scalp/calvarium centered on the vitamin E capsule. An 8 cm^3^ voxel (2 cm × 2 cm × 2 cm) was placed on one of the oblique localizer slices such that: (1) the voxel was centered on the vitamin E capsule; and (2) the voxel was positioned as superficially as possible on the left precentral gyrus such that the outer margin of the voxel did not enter the calvarium. This region encompassed the hand knob of the precentral gyrus (Brodmann area 4) that was presumably interrogated with TMS. The ACC voxel was positioned as previously described (Croarkin et al., 2016), encompassing the pregenual ACC of both hemispheres (Brodmann areas 24a, 24b and 32). A TE-optimized PRESS sequence (PROBE-P, TR = 2000 ms, TE = 80 ms, 128 acquisitions, 5000 Hz bandwidth, 2048 points) was used to obtain spectroscopic data for both voxel locations.

#### Reconstruction and Quantification of Spectra

After acquisition, the spectroscopic imaging data were transferred to a UNIX workstation running SAGE-IDL (GE Medical Systems, Inc., Waukesha, WI, USA). Visual verification of data integrity was performed, with scans demonstrating significant artifact being excluded from analysis. Quantitative analysis of metabolites was performed with LCModel software (Provencher, 1993), with the basis set provided by the vendor. A Cramér-Rao lower bound of 20 (for measurement of error) was used, and data with bounds higher than 20 were excluded (Kreis, 2016).

#### Tissue Segmentation and CSF Correction

Anatomical image data was segmented into gray matter (GM), white matter (WM), and CSF as previously described (Port et al., 2008; Croarkin et al., 2016). Briefly, voxel locations were superimposed upon the segmented anatomical data, and the fractions of GM, WM, and CSF were summed for each imaging pixel in the spectroscopy voxel. The fraction of CSF (F_CSF_) in each spectroscopy voxel was computed and used to correct metabolite concentrations:

(1) $${\left[\text{M}\right]}_{\text{Corrected}}\;=\;{\left[\text{M}\right]}_{\text{Measured}}\times \left(\frac{1}{1-{\text{F}}_{\text{CSF}}}\right)$$

CSF-corrected metabolite concentrations are reported in “institutional units.” All statistical analyses were performed with CSF-corrected concentrations of metabolites. ^1^H-MRS spectra yielded CSF-corrected concentrations of the following metabolites in the ACC and left motor cortex voxels: creatine (Cr), choline (Cho), *n*-acetylaspartate (NAA), glutamate (Glu), and glutamate + glutamine (Glx).

### Statistical Analysis

To assess relationships between TMS measures and glutamatergic metabolite concentrations, correlation coefficients were calculated for each TMS measure and [Glx] in each voxel, with an α level of 0.05 (two-tailed) for significance. Distributions of TMS measures of cortical excitability and inhibition and ^1^H-MRS-measured Glx concentrations in each voxel were assessed for normality using the Shapiro-Wilk test. [Glx] in both voxels and all TMS neurophysiologic measures demonstrated normal distributions by the Shapiro-Wilk test, with the exception of the MEP amplitudes in the 2-ms SICI paradigm (*W* = 0.8507, *p* = 0.0227) and the 20-ms ICF paradigm (*W* = 0.7281, *p* = 0.0007). However, visual inspection of histograms and Q-Q plots revealed substantial skewness in many of the distributions. In light of the small sample size and low power to reject a null hypothesis of normality, we elected to report both the parametric Pearson product-moment correlation coefficient (*r*) and the non-parametric Spearman rank-order correlation coefficient (*ρ*) for all correlations between TMS measures and [Glx]. As this was an exploratory study aimed at detecting signals of relationships of interest for hypothesis generation, uncorrected *p*-values are reported and interpreted. However, for the interested reader, adjustment of *p*-values for multiple comparisons using the false discovery rate (FDR) method (Benjamini and Hochberg, 1995) was performed, and these are reported as well. JMP 10.0.0 software (SAS Institute, Inc., Cary, NC, USA) was utilized for all statistical analyses.

## Results

### Participants

Twenty-four adolescents (16 female, 8 male) with a mean age of 15.2 ± 1.8 years enrolled in the study. Of these, 14 participants (11 female, 3 male; mean age 14.7 ± 1.9 years) completed ^1^H-MRS procedures. Participant demographics, including mean depression severity scores on the CDRS-R and QIDS-A_17_-SR, are reported in Table 1. Fifteen participants were taking psychotropic medications at the time of the study; this included 14 taking selective serotonin reuptake inhibitors, two taking psychostimulants (which were held on days of TMS and ^1^H-MRS testing), one taking an atypical antipsychotic agent, and two taking sedative-hypnotics. For a full list of participants’ psychotropic medications and doses at the time of the study, see Supplementary Table S1.

**Table 1:** **Participant demographics**.

	All participants	^1^H-MRS participants
Sex		
Female	*n* = 16	*n* = 11
Male	*n* = 8	*n* = 3
Age	15.2 ± 1.8 years	14.7 ± 1.9 years
Ethnicity		
African-American	*n* = 2	*n* = 1
African-American/Caucasian	*n* = 1	*n* = 1
Asian	*n* = 2	*n* = 1
Caucasian	*n* = 17	*n* = 9
Hispanic	*n* = 1	*n* = 1
Native American	*n* = 1	*n* = 1
CDRS-R score	39.2 ± 13.0	41.5 ± 14.4
QIDS-A_17_-SR score	11.5 ± 5.2	11.9 ± 5.9

### TMS and MRS Measures

Mean values and standard deviations for the TMS measures (RMT, CSP, SICI-2, SICI-4, ICF-10, ICF-15, and ICF-20) are reported in Table 2. In the left primary motor cortex, mean [Glx] obtained via ^1^H-MRS was 11.35 ± 1.42 institutional units. In the medial ACC, mean [Glx] was 18.45 ± 5.03. Other neural metabolite concentrations obtained via ^1^H-MRS are reported in Supplementary Materials (Table S2).

**Table 2:** **Excitatory and inhibitory TMS measures**.

	All participants	^1^H-MRS participants
RMT	60.79 ± 11.94	60.43 ± 11.75
CSP	0.190 ± 0.051 s	0.194 ± 0.061 s
SICI-2	0.440 ± 0.314	0.495 ± 0.358
SICI-4	0.578 ± 0.296	0.474 ± 0.279
ICF-10	1.568 ± 0.465	1.455 ± 0.367
ICF-15	1.765 ± 0.447	1.681 ± 0.413
ICF-20	2.299 ± 0.699	2.472 ± 0.698

*Abbreviations: RMT, resting motor threshold; CSP, cortical silent period; SICI-2, -4, short-interval intracortical inhibition at 2-ms and 4-ms interstimulus intervals, respectively; ICF-10, -15, -20, intracortical facilitation at 10-ms, 15-ms and −20-ms interstimulus intervals, respectively. RMT is expressed as a percentage of maximal device output. MEP amplitudes in the SICI and ICF paradigms are expressed as ratios to the mean unconditioned stimulus amplitude*.

### Cortical Excitability and Inhibition—Cortical Glutamate Relationships

#### Left Primary Motor Cortex

There was a significant positive correlation between RMT and left motor cortex [Glx] (*r* = 0.6533, *p* = 0.0113; *ρ* = 0.6300, *p* = 0.0158). Other relationships between TMS measures and left motor cortex [Glx] were not significant (Table 3).

**Table 3:** **Correlations between ^1^H-MRS-measured glutamatergic metabolite concentrations and TMS measures of cortical excitability and inhibition**.

	Left primary motor cortex [Glx]						Medial anterior cingulate cortex [Glx]					
	Pearson *r*	*p*	*p*_FDR_	Spearman *ρ*	*p*	*p*_FDR_	Pearson *r*	*p*	*p*_FDR_	Spearman *ρ*	*p*	*p*_FDR_
RMT	0.6533	0.0113*	0.0791	0.6300	0.0158*	0.1827	−0.4027	0.1943	0.3022	−0.5149	0.0867	0.2616
CSP	−0.0635	0.8293	0.8931	−0.1824	0.5325	0.6777	−0.1373	0.6704	0.8532	−0.2657	0.4038	0.5653
SICI-2	0.0990	0.7363	0.8590	0.0725	0.8054	0.8403	0.4414	0.1508	0.2639	0.4615	0.1309	0.2618
SICI-4	0.5093	0.0629	0.2639	0.4593	0.0985	0.2616	−0.1504	0.6408	0.8532	−0.1469	0.6488	0.7569
ICF-10	−0.4123	0.1429	0.2639	−0.4901	0.0752	0.2616	0.4742	0.1193	0.2639	0.6364	0.0261*	0.1827
ICF-15	0.0045	0.9879	0.9879	−0.0593	0.8403	0.8403	−0.4753	0.1184	0.2639	−0.4825	0.1121	0.2616
ICF-20	−0.4704	0.0896	0.2639	−0.3890	0.1692	0.2961	0.7201	0.0083*	0.0791	0.4056	0.1908	0.2968

*p < 0.05. Abbreviations: [Glx], glutamate+glutamine; RMT, resting motor threshold; CSP, cortical silent period; SICI-2, -4, short-interval intracortical inhibition at 2-ms and 4-ms interstimulus intervals, respectively; ICF-10, -15, -20, intracortical facilitation at 10-ms, 15-ms and 20-ms interstimulus intervals, respectively; *p_FDR_*, *p*-value corrected for multiple comparisons by the false discovery rate procedure.

#### Medial Anterior Cingulate Cortex

The MEP amplitude in the 10-ms ICF paradigm (ICF-10) had a significant positive relationship with [Glx] (*ρ* = 0.6364, *p* = 0.0261) in the medial ACC. Additionally, the MEP amplitude in the 20-ms ICF paradigm (ICF-20) demonstrated a significant positive correlation with [Glx] (*r* = 0.7201, *p* = 0.0083). Correlations between ACC [Glx] and other TMS measures were not significant (Table 3).

## Discussion

This was an exploratory study investigating the potential link between glutamatergic neurochemistry and excitatory and inhibitory neurophysiology. To our knowledge, this was the first study to examine relationships between ^1^H-MRS-measured concentrations of neural metabolites and TMS measures of cortical excitation and inhibition in a psychiatric population, as well as the first to characterize these relationships in adolescents. Additionally, unlike prior TMS-MRS studies, neural metabolite concentration values were corrected to CSF. This eliminates the potential distortion that may be caused by correcting values to creatine, whose concentration may be altered in pathological conditions (Maddock and Buonocore, 2012) and indeed has been found to differ in adults with mood disorders (Frye et al., 2007; Port et al., 2008) as well as adolescents with MDD (Gabbay et al., 2007) and other psychopathology (Mirza et al., 2006).

### Motor Cortex Glutamate and Cortical Excitability

The question of whether properties of TMS-induced MEPs originating in the primary motor cortex correspond to neurotransmitter levels in the same region of cortex is intuitive, and indeed these relationships have been the focus of two prior studies in healthy adults and one study in adults with mild brain injury. Stagg et al. (2011) investigated the relationships of GABA and glutamate concentrations in the motor cortex to various TMS measures of excitability and inhibition. The authors found that the MEP I-O curve, considered to be a global measure of excitability, was positively correlated with both motor cortex glutamate and motor cortex GABA concentrations; however, the MEP I-O—glutamate relationship did not survive correction for GABA concentration. In another study, Tremblay et al. (2013) noted a significant positive association between the CSP duration and motor cortex [Glx], a relationship that remained significant after controlling for GABA concentration. No significant correlations between ^1^H-MRS-measured [GABA] and CSP, SICI, or LICI were found. In another study, Tremblay et al. (2014) found a correlation between LICI and motor cortex [GABA] in young adult athletes with histories of concussion, a relationship that was absent in healthy control comparators, suggesting potential alterations in GABA-mediated inhibitory physiology due to brain pathology.

In the present study, we found a strong correlation between the RMT and motor cortex [Glx]. Interestingly, the relationship was positive (i.e., higher levels of [Glx] corresponded to higher thresholds for evoked potentials), which is somewhat counterintuitive and also differs from the direction of the relationship between RMT and ACC glutamate concentration (which did not quite meet the threshold for significance). Numerous factors could account for this. The physiology of the RMT is complex; both cortico-cortical and corticospinal neurons are involved (Sandbrink, 2008), and both voltage-gated sodium channels and glutamatergic receptors have been demonstrated to mediate the threshold level (Ziemann et al., 2015). The RMT may best be described as a global index of cortical and spinal excitability that has many inputs, including glutamate-mediated neurotransmission. Thus, it perhaps would be overly simplistic to assume that an increase in glutamate necessarily leads to a decrease in RMT, as autoregulatory feedback loops and other mechanisms may mediate the effect of local glutamate concentration on the RMT. Furthermore, concentrations of glutamate or glutamate + glutamine in a region of cortex may not be particularly accurate indices of the pool of glutamate available for excitatory functions. Vesicular glutamate is not well detected by ^1^H-MRS, and the concentrations measured by the current spectroscopic techniques may better represent the total neuronal and glial stores of glutamate and glutamine available for both synaptic and metabolic functions (Yüksel and Öngür, 2010; Maddock and Buonocore, 2012). Magnetic fields greater than the 3 T employed for ^1^H-MRS in this study are necessary to separate signals for glutamine and glutamate reliably. Additionally, there is growing recognition of the role of neuroactive chemicals in the extracellular matrix on intercellular communication in the brain (Marcoli et al., 2015). Nevertheless, it is notable that glutamatergic metabolites in the primary motor cortex demonstrated associations with the RMT, and further research is necessary to characterize the mechanisms by which glutamate mediates this aspect of excitatory physiology.

### ACC Glutamate and Cortical Excitability

The few prior studies that examined relationships between TMS measures of cortical excitability and inhibition and cortical glutamatergic concentrations selected ^1^H-MRS voxels only in the motor cortex. There is robust evidence from prior ^1^H-MRS studies that glutamate metabolism in the ACC is disrupted in youth with MDD (Mirza et al., 2004; Rosenberg et al., 2004, 2005; Kondo et al., 2011), but it is not clear what effect this has on physiologic functions mediated by the motor cortex. Consequently, we investigated the relationships between ACC glutamate concentrations and TMS measures of cortical excitability and inhibition.

ACC [Glx] did not demonstrate significant correlations with inhibitory measures (CSP, SICI). However, [Glx] did demonstrate a strong positive correlation with MEP amplitude in the 10-ms ICF paradigm (in nonparametric correlations) and in the 20-ms paradigm (parametric), with higher concentrations of glutamate in the ACC corresponding to greater facilitation of the MEP. One should be cautious in assuming that increases in excitatory neurotransmitters in one area of cortex necessarily lead to increased excitatory physiology in another region. Nonetheless, these findings suggest that increased glutamate (or increased glutamate + glutamine stores) in the ACC may have downstream effects on the excitatory physiology mediated by the motor cortex, which is plausible in light of the dense connectivity between these two cortical regions (Paus, 2001). Replication, particularly in healthy control adolescents, would help to establish such a relationship. It is unclear why the nonsignificant ICF-ACC glutamate correlation was negative at the 15-ms ISI, but this could be related to subtle differences in the physiology of facilitatory responses at different ISIs, something that has been observed with GABA-mediated SICI paradigms (Fisher et al., 2002; Ilić et al., 2002; Hanajima et al., 2003; Stagg et al., 2011).

In contrast to these ICF findings, the correlation between ACC glutamate and the RMT, which did not quite reach significance, was negative. This differs from the statistically significant, positive correlation between the RMT and [Glx] in the primary motor cortex. The negative relationship observed between glutamate concentrations in the ACC and the RMT could be related to glutamate-mediated regulatory circuits between the ACC and the motor cortex. As noted above, the physiology of the RMT involves many elements, notably the voltage-gated sodium channels, and it is possible that within the motor cortex these dominate the excitability responsible for motor threshold, with glutamate playing a lesser role. Future studies investigating the relationship between ACC glutamate and TMS measures of cortical excitability in healthy adolescent populations may help to understand the excitatory physiology of ACC-motor cortex connections further.

### Limitations

The current study has several notable limitations. First, the sample size was small, particularly the subset that completed both TMS and ^1^H-MRS procedures. Although similar in size to the few previous TMS-MRS studies, this limited our statistical approaches. Correlations that were significant based on raw *p*-values did not survive correction for multiple comparisons, which is unsurprising in light of the number of correlations performed and the small number of participants. Thus, the findings should be interpreted with caution. Larger studies with greater power are warranted, particularly as this could permit controlling for potential confounds. Additionally, the sample did not include healthy controls. In the absence of data from healthy youth, it is not possible to determine to what extent depression may impact these findings, and future studies should endeavor to compare healthy participants and participants with psychopathology. The participants in this study were not medication-naïve, unlike many of the previous studies utilizing ^1^H-MRS or TMS methods. Although concurrent psychotropic medications or history of pharmacologic interventions do pose potential confounds, the sample of our study is potentially more representative of populations seen in typical clinical practice. An additional potential confound is the impact of the menstrual cycle on both TMS and ^1^H-MRS data, particularly as the sample was comprised predominantly of female adolescents. Prior TMS research has shown cortical inhibition to increase during the luteal phase in healthy women (Smith et al., 1999, 2003) but to decrease in women with premenstrual dysphoria (Smith et al., 2003), and ^1^H-MRS studies have shown alterations in cortical GABA levels across the menstrual cycle (Epperson et al., 2002, 2005; Harada et al., 2011). While menstrual phase and sex hormone levels were not assessed in the female adolescent participants in the present study, future investigations should consider the value of additional measures to assess and control for sex- and menstrual-related effects.

Previous research has established that significant changes occur in excitatory and inhibitory circuitry systems during the childhood and adolescent years. In early life, expression of various subunits that compose GABA_A_ receptors has been found to change over time (Duncan et al., 2010), while GABA_A_ receptor density changes at differing rates in various brain regions into adulthood (Chugani et al., 2001). GABA receptors also show paradoxically excitatory activity in early childhood, with a gradual shift toward inhibition with age (Rakhade and Jensen, 2009). Glutamatergic neurotransmission also undergoes developmental shifts, including alterations in expression of glutamatergic receptor types, as well as modifications in receptor subunit composition affecting ion permeability (Silverstein and Jensen, 2007). In the TMS literature, one of the most consistent age-related findings is that the RMT diminishes with increasing age, both in neurologically- and psychiatrically-healthy individuals (Nezu et al., 1997; Heinen et al., 1998; Moll et al., 1999; Garvey et al., 2003; Mall et al., 2004; Gilbert et al., 2011) as well as those with conditions such as attention-deficit/hyperactivity disorder (Gilbert et al., 2011), Tourette syndrome (Moll et al., 2006), and MDD (Croarkin et al., 2014). The impact of age on inhibitory TMS measures is less clear, with some studies finding CSP duration (Heinen et al., 1998; Moll et al., 1999), SICI (Mall et al., 2004), and LICI (Croarkin et al., 2014) to increase with age, while others have found no significant relationship with age for CSP (Garvey et al., 2003; Gilbert et al., 2011; Croarkin et al., 2014) or SICI (Moll et al., 1999; Gilbert et al., 2004, 2011; Croarkin et al., 2014). The statistical methods of the present study do not permit controlling for age; however, future studies utilizing neurophysiologic TMS in children and adolescents could consider regression analyses with age as a covariate. Further research is warranted to characterize more conclusively the developmental trajectories of TMS-measured cortical excitability and inhibition in the context of MDD.

In the processing of ^1^H-MRS data, there are inherent challenges in separating signals for Glu, Gln and GABA. Our ^1^H-MRS procedures did not permit identification of a distinct GABA signal, in contrast to other TMS-MRS studies (Stagg et al., 2011; Tremblay et al., 2013, 2014). However, there is significant interaction between GABAergic and glutamatergic systems, both at the level of their neurochemical pathways and in the excitatory-inhibitory balance achieved by circuits of GABAergic and glutamatergic neurons. Additionally, the interpretation of glutamatergic metabolite concentration data is difficult, as ^1^H-MRS-measured concentrations reflect the combined neuronal and glial stores of glutamate and glutamine, and also are believed to represent primarily cytoplasmic (as opposed to mitochondrial or vesicular) concentrations (Maddock and Buonocore, 2012). While total quantities of glutamatergic metabolites would be expected to influence glutamate-mediated (and GABA-mediated) processes, neuronal vesicular glutamate concentrations may be more immediately related to the neurotransmission underlying the excitatory and inhibitory phenomena measured by TMS. Moreover, the spectroscopic data were obtained at a single point in time. This is relevant in light of evidence from human ^1^H-MRS studies in which glutamatergic metabolites have been demonstrated to fluctuate in response to sensory stimuli (Mullins et al., 2005; Mangia et al., 2007; Gussew et al., 2010) and physical activity (Maddock et al., 2011), suggesting that glutamate concentrations are state-dependent and dynamic (Maddock and Buonocore, 2012). Although the ^1^H-MRS data in the present study were obtained on the same day as TMS testing, they were not concurrent with the excitatory and inhibitory processes induced by TMS.

## Conclusion

The preliminary evidence from this small study supports the role of glutamate in cortical excitatory processes that are measured by TMS. To our knowledge, this was the first study to utilize the combination of TMS and ^1^H-MRS in a psychiatric population as well as the first to do so with adolescents. Further research aimed at examining the relationships between glutamatergic metabolite concentrations measured by MRS and excitatory and inhibitory physiology measured by TMS is warranted. Combined TMS-MRS methods have potential utility for investigating developmental changes in excitatory and inhibitory processes in healthy youth, studying the pathophysiology of common neurodevelopmental psychiatric conditions such as MDD, and ultimately for the development of biomarkers in children and adolescents.

## Author Contributions

CPL, JDP, MAF, JLVV, SHA, MMH, ZJD, and PEC contributed to the design of the study and interpretation of data. CPL, JLVV, and PEC acquired the data; JDP processed and analyzed the spectroscopic data; CPL and PEC processed transcranial magnetic stimulation data; CPL and PEC completed statistical analyses; CPL, JDP, MAF, JLVV, SHA, MMH, ZJD, and PEC drafted, revised, critically reviewed and approved the final submitted draft of the manuscript.

## Conflict of Interest Statement

MAF has had grant support from Assurex, Myriad, and Pfizer and has served as an unpaid consultant for Janssen Global Services LLC, Mitsubishi Tanabe Pharma Corporation, Myriad, Sunovion, Supernus Pharmaceuticals, and Teva Pharmaceuticals. MMH has received grant support from Alkermes, Assurex, Avanir, Cyberonics, Brainsway, MagStim, NeoSync, Neuronetics, the Stanley Foundation, and St. Jude Medical (Advanced Neuromodulation Systems). He has received research and equipment in-kind support for an investigator-initiated study through Brainsway, and a travel allowance through Merck. ZJD received research and equipment in-kind support for an investigator-initiated study through Brainsway and a travel allowance through Merck. He has received speaker funding through Sepracor and AstraZeneca, served on advisory boards for Hoffmann–LaRoche Limited and Merck, and received speaker support from Eli Lilly. PEC has received investigator-initiated grant support through Pfizer and in-kind support from Assurex (supplies and genotyping for an investigator-initiated study) and Neuronetics (supplies for investigator-initiated studies). He is a site primary investigator for a multicenter trial sponsored by Neuronetics. CPL is a site co-investigator for a multicenter trial sponsored by Neuronetics. JLVV is a site co-investigator for a multicenter trial sponsored by Neuronetics and has in-kind support from Assurex. The remaining authors declare no conflicts of interest.
