# Supplementary material for: An Exploratory Study of Spectroscopic Glutamatergic Correlates of Cortical Excitability in Depressed Adolescents
Source: Front Neural Circuits. 2016 Nov 29;10:98. doi: 10.3389/fncir.2016.00098 (PMC5127083; doi:10.3389/fncir.2016.00098)
Supplement: Supplementary file 1 [file Table_1.docx]

Table 1s. Psychotropic medications at time of TMS and ^1^H-MRS

| Participant | Medications |
| --- | --- |
| 1* |  |
| 2* | fluoxetine 40 mg |
| 3* |  |
| 4* | fluoxetine 20 mg |
| 5* | fluoxetine 30 mg |
| 6* |  |
| 7* | escitalopram 10 mg |
| 8 |  |
| 9* |  |
| 10* |  |
| 11* |  |
| 12* |  |
| 13* | escitalopram 10 mg |
| 14 | fluoxetine 60 mg, methylphenidate 60 mg^†^, trazodone 75-100 mg |
| 15 | escitalopram 20 mg |
| 16* | escitalopram 10 mg |
| 17 | fluoxetine 20 mg |
| 18 | escitalopram 10 mg |
| 19 | fluoxetine 10 mg |
| 20* | sertraline 100 mg |
| 21 | methylphenidate 18 mg^†^ |
| 22 | sertraline 200 mg, aripiprazole 2 mg |
| 23 | fluoxetine 20 mg, melatonin 3 mg |
| 24 |  |
| * Completed ^1^H-MRS. ^†^ Psychostimulant medications were held on days of TMS/^1^H-MRS testing. Total daily doses reported. | |
