# Supplementary material for: An Exploratory Study of Spectroscopic Glutamatergic Correlates of Cortical Excitability in Depressed Adolescents
Source: Front Neural Circuits. 2016 Nov 29;10:98. doi: 10.3389/fncir.2016.00098 (PMC5127083; doi:10.3389/fncir.2016.00098)
Supplement: Supplementary file 2 [file Table_2.docx]

Table 2s. Neural metabolite concentrations measured by ^1^H- MRS

|  | Left Primary Motor Cortex |  | Medial Anterior Cingulate Cortex |
| --- | --- | --- | --- |
| [Cr] | 11.10 ± 0.95 |  | 14.20 ± 1.30 |
| [Cho] | 2.55 ± 0.37 |  | 3.42 ± 0.42 |
| [NAA] | 20.36 ± 1.37 |  | 19.41 ± 1.66 |
| [Glu] | 9.64 ± 1.27 |  | 14.93 ± 4.18 |
| [Glx] | 11.35 ± 1.42 |  | 18.45 ± 5.03 |
| Abbreviations: [Cr], creatine; [Cho], choline; [NAA], *n*-acetylaspartate; [Glu], glutamate; [Glx], glutamate+glutamine.  Concentrations expressed in institutional units. | | | |
